# Implementation of a Locally Deployed Qwen2.5-7B-Instruct Pipeline for Structured Classification of Elbow MRI Reports

**DOI:** 10.7759/cureus.114918

**Published:** 2026-08-21

**Authors:** Kian A Huang, Sonia V Prakash, David Samvelian, Neelesh S Prakash

**Affiliations:** 1 Radiology, University of South Florida (USF) Health Morsani College of Medicine, Tampa, USA

**Keywords:** elbow mri, large language models (llm), radiology reports, report mining, text extraction

## Abstract

Large language models (LLMs) are increasingly used for radiology report extraction, but many published applications lack the implementation details needed for reproducibility. This technical report provides a complete implementation-level description of a locally deployed LLM pipeline for structured classification of radiology reports. We describe a report classification system built around Qwen2.5-7B-Instruct, an open-weight instruction-tuned LLM served through a local Ollama runtime rather than a cloud application programming interface, and applied to a corpus of 10,222 elbow magnetic resonance imaging (MRI) reports. The report details the complete processing workflow, including report ingestion and column resolution, cohort-defining pattern matching, batch construction and threaded execution, the structured prompt used to generate two binary classification labels per report, output parsing, single-retry failure handling, and statistical routines used to summarize extracted data. To document the implementation comprehensively, we also describe the creation of an independently reviewed reference set of 400 reports, including reviewer blinding and file-separation procedures, as well as configuration factors that may influence reproducibility in locally deployed LLM workflows, including model quantization, sampling temperature, runtime version, and practical approaches for controlling these variables. This report is intended as a standalone technical reference that enables readers to understand, reproduce, evaluate, or adapt the described pipeline for related musculoskeletal or radiology report extraction tasks without requiring access to the original codebase.

## Introduction

Large language models (LLMs) are increasingly being applied to the automated extraction and classification of information from free-text radiology reports, offering the potential to accelerate research cohort identification, quality improvement initiatives, and large-scale analysis of imaging data [1,2]. As their use in radiology has expanded, they have been applied to a growing range of text-mining tasks, from structuring narrative findings into standardized fields to identifying clinically relevant imaging features for downstream analysis [1,2]. However, the performance and reproducibility of these systems are highly dependent on implementation details. Factors including the exact wording of the prompt, handling of negation, sampling parameters that govern output variability, and the quantization level of an open-weight model can each materially influence classification behavior [3-6]. Because these configuration choices are often incompletely described in published work, reported performance can be difficult for other groups to interpret, reproduce, evaluate, or build upon.

These challenges are further amplified when using locally deployed, open-weight models. Cloud-hosted application programming interfaces generally provide a standardized software environment with fixed model versions and default inference settings throughout a study. In contrast, locally deployed models introduce additional implementation variables, including the runtime version, the specific quantized weight file associated with a model, and the underlying hardware and software environment, each of which may affect model behavior but is rarely reported in sufficient detail. Despite this added complexity, local deployment is often the preferred or only practical option for institutions that cannot transmit protected health information to external services, and open-weight models have demonstrated competitive performance with closed, cloud-hosted alternatives for comparable radiology report extraction tasks [7]. Consequently, comprehensive technical documentation of these implementation choices is essential for reproducibility and for enabling other investigators to adapt similar workflows.

This technical report provides a complete implementation-level description of a pipeline developed to classify a large corpus of elbow MRI reports for two tendon pathologies. Rather than evaluating model performance or presenting a clinical investigation, the report documents the full software environment, prompt specification, output parsing and error-handling procedures, concurrent execution strategy, and construction of an independently reviewed human-reference dataset. The objective is to provide sufficient technical detail for readers to understand, reproduce, critically evaluate, or adapt the pipeline for related radiology report extraction tasks without requiring access to the original source code.

## Technical report

Data source and organization

The source dataset consisted of non-contrast upper extremity MRI reports (n = 51,169) obtained from a private teleradiology dataset provided by Radiology Partners (Austin, Texas, United States) of reports collected from examinations performed between 2025 and 2026. The dataset was used for technical implementation and demonstration of the automated classification workflow, including report ingestion, cohort identification, model inference, structured output generation, and downstream data processing. Because this report focuses on pipeline implementation rather than clinical validation, the generated labels are presented as automated pipeline outputs and not as reference-standard determinations of pathology. Formal evaluation against expert annotation was outside the scope of this work.

Configuration values are given here as they appear in the underlying implementation rather than rounded or generalized, on the principle that a technical description of a pipeline is only useful if it is exact.

Software and serving environment

The pipeline was implemented in Python 3 (Python Software Foundation, Wilmington, Delaware, United States) and depends on pandas and numpy for data handling, the requests library for communicating with the model server, tqdm for progress tracking during batch execution, and scipy.stats together with statsmodels for the statistical routines described below. The language model itself is not called through a hosted application programming interface; instead, it is served locally by Ollama, a self-hosted model runtime [8], and reached through a fixed local network endpoint. Table 1 lists the concrete runtime parameters used for the classification run described in this report.

**Table 1:** Runtime configuration of the classification pipeline GPU: graphics processing unit

Parameter	Value
Model name	Qwen2.5:7b-instruct
Virtual environment	Ollama
Inference hardware	Local GPU-accelerated host
Per-request timeout	120 seconds
Failed-request retry policy	Up to three attempts, with a two-second pause between attempts
Reports per prompt (batch size)	Three
Concurrent workers	Three (thread pool executor)
Malformed-output retry	One retry, with an appended formatting reminder
Output structure	A list of records, each containing a report identifier, a distal biceps pathology label, and a common extensor pathology label

Model documentation

Qwen2.5-7B-Instruct is the seven-billion-parameter, instruction-tuned variant of the Qwen2.5 language model family, released with openly available weights [9]. It was selected for this pipeline over larger models in the same family primarily for deployability: a seven-billion-parameter model can be served locally on a single consumer or workstation-grade GPU (graphics processing unit) with acceptable latency, whereas the larger variants in the same family typically require multi-GPU or data-center-class hardware to serve at comparable speed [10]. This deployability was the determining factor given the requirement that report text never leave local infrastructure. No fine-tuning or parameter adaptation was applied; the model was used strictly through prompting, with all task-specific behavior encoded in the prompt described below rather than in model weights.

Two aspects of the deployment are relevant to interpreting its output and are worth stating explicitly because they are not fixed by the model tag alone. First, Ollama distributes most models in a quantized form by default, and the specific quantization level associated with a given tag can change between Ollama releases [11]. Second, the sampling temperature and related decoding parameters, such as top-p, were left at Ollama's defaults for this model rather than being explicitly set in the request payload; because the classification task is closer to structured extraction than open-ended generation, output was empirically stable across repeated runs on the same report, but a deployment requiring strict determinism should set these parameters explicitly rather than relying on defaults.

Report ingestion and cohort definition

Report data were read from a tabular export using pandas, and column headers were stripped of surrounding whitespace on load. The column holding report text was located programmatically, by scanning for any column name containing the word report, rather than referenced by a fixed name, which allowed the ingestion step to tolerate minor differences between export files. Rows lacking report text were dropped, the retained text was coerced to string type and whitespace-trimmed, and the working table's row index was reset immediately afterward. The index was reset immediately before inference because downstream identifiers are based on the row index at that stage. This ensured consistent report-to-classification mapping and prevented alignment errors introduced by indices carried over from earlier filtering steps.

The elbow-related subset of the broader report corpus was identified from a procedure-description field using a case-insensitive text-matching rule that flagged any report whose procedure description referenced an elbow MRI, in either word order, the elbow generally, the upper extremity, the humerus, or the forearm, applied without any additional filtering on pathology content. This rule was written intentionally broadly, favoring recall over precision at the cohort-definition stage, so that the classification step, rather than the cohort-selection step, would be responsible for determining pathology status. Of the 51,169 reports in the source export, 10,222 matched this rule and constituted the working cohort for the remainder of the pipeline.

Batch construction and concurrent execution

Reports were grouped into fixed batches of three consecutive rows, and each batch was rendered into a single prompt in which every report was preceded by a line identifying it by its integer row index, so that the model's response could later be mapped back to a specific source report. Batches were submitted concurrently through a three-worker thread pool, with results collected as each batch finished rather than in submission order. Immediately after all batches completed, the raw, unaggregated model output was saved to a checkpoint file on disk before any parsing, merging, or statistical computation took place, so that a fault in a later stage would not require repeating the inference step.

Prompt specification

A single prompt template was applied uniformly to every batch. The template was organized into six labeled components, summarized in Table 2 and detailed in the terminology that follows.

**Table 2:** Structural components of the classification prompt

Component	Function
Output-format rules	Restricts the response to a single, unadorned structured array, with no narrative commentary, double-quoted fields, and lowercase boolean values, each record addressed by exact report identifier
Variable 1 criteria	Enumerates the terminology that should be treated as positive evidence of distal biceps pathology
Variable 2 criteria	Enumerates the terminology that should be treated as positive evidence of common extensor, or lateral epicondylar, pathology
Negation handling	Specifies phrasing, such as intact, unremarkable, or no evidence of, that should force a negative label for a variable unless overridden by an explicit positive statement elsewhere in the same report
Interpretive constraints	General instructions to read Findings and Impression together, avoid inferring unstated pathology, default to negative when uncertain, weight current imaging over historical symptom language, and count prior surgery as positive only when a current abnormality is also described
Worked example	A single minimal example of the required output object, given to anchor the model's output format

The complete positive-evidence terminology for each classification variable is reproduced below. The exact lexical coverage is a primary determinant of pipeline sensitivity and is therefore essential for reproducibility and any attempt at replication.

For distal biceps pathology, positive evidence comprised tendinosis, tendinopathy, tendinitis, degeneration, degenerative change, fraying, or general abnormality of the distal biceps tendon; partial-, low-, high-grade, split-, or full-thickness tears and rupture; attenuation, thickening, edema, strain, or fiber disruption of the tendon; insertional pathology, including insertional tendinopathy, insertional degeneration, insertional tearing, or insertional fraying; bicipitoradial bursitis when associated with distal biceps disease or reactive edema surrounding the tendon; and abnormal tendon signal, tendon heterogeneity, tendon irregularity, or tendon delamination.

For common extensor (lateral epicondylar) pathology, positive evidence comprised lateral epicondylitis or lateral epicondylopathy; common extensor tendinosis, tendinopathy, tendinitis, degeneration, degenerative change, fraying, or general abnormality; partial-, low-, high-grade, split-, or full-thickness tears, rupture, fiber disruption, tendon heterogeneity, or irregularity; extensor carpi radialis brevis pathology, tendinosis, tear, or degeneration; the colloquial term tennis elbow, lateral elbow tendinopathy, or lateral condylar tendinopathy; and extensor origin degeneration, tearing, or tendinopathy.

For both variables, the prompt directed the model to count chronic, postoperative, degenerative, mild, low-grade, and healed disease as positive whenever it was currently documented on the MRI, rather than excluding lower-acuity language from consideration. A shared negation rule set applied to both variables: a report was labeled negative for a given structure if it contained phrasing such as no tear, intact, normal, unremarkable, without abnormality, no evidence of, negative for, or an explicit tendon-intact statement for that structure, unless that negation was contradicted by an explicit positive statement elsewhere in the same report.

Response parsing and failure handling

Model output was reduced to structured data through a fixed procedure applied independently to every batch. The first and last bracket characters in the raw response text were located, and the text between them was taken as the candidate output payload; this candidate was then parsed with a standard structured-data decoder, and if parsing succeeded, the result was accepted and passed to the merge step described below. If parsing failed, the same batch was resubmitted exactly once with an additional line appended to the prompt reiterating that only a valid structured array should be returned; if the retried response also failed to parse, the batch was recorded as failed and its output discarded, with no further retry attempted. For every successfully parsed record, its report identifier was cast to an integer; if this cast failed, that individual record was discarded, with a logged warning, rather than allowing the error to propagate downstream. Accepted records were then merged into the full cohort table by report identifier. Before merging the accepted inference results, each report record was initialized with negative values for both classification fields. During the merge, reports with successfully parsed identifiers had these initialized values replaced by the corresponding model outputs. Reports whose identifiers were unavailable because of an unrecoverable batch failure or discarded record retained their initialized values, ensuring that every input report remained represented in the final dataset and preserving one-to-one correspondence between the source reports and output table. This initialization serves as an implementation safeguard for dataset integrity and should not be interpreted as a validated classification strategy or recommendation for clinical deployment.

Cohort-level statistical routines

Table 3 lists the statistical procedures implemented downstream of the classification stage, together with the library function used for each and its role in summarizing pipeline output. These routines are included to document the post-processing functionality implemented within the pipeline and to illustrate how extracted labels can be summarized at the cohort level. They are not presented as hypothesis-driven analyses or as evidence of clinically meaningful associations between the classified variables. Accordingly, the resulting prevalence and association estimates are not reported here, as the purpose of this report is to document the implementation and statistical workflow rather than to present or interpret clinical findings.

**Table 3:** Statistical routines applied to pipeline output at the cohort level

Statistic	Implementation	Purpose
Wilson score interval	statsmodels.stats.proportion.proportion_confint	95% confidence bound on each variable's cohort-wide prevalence
Chi-square test of independence	scipy.stats.chi2_contingency	Tests association between the two classified variables on the full contingency table
Odds ratio (continuity-corrected)	statsmodels Table2x2, applied after a Haldane-Anscombe 0.5-cell correction	Yields a finite odds ratio and confidence interval even when a cell count is small
Fisher's exact test	scipy.stats.fisher_exact	Cross-checks the chi-square association result on the uncorrected table
Relative risk	Computed directly from contingency-table cell counts	Expresses co-occurrence as a prevalence ratio between the two variables
Phi coefficient	Square root of the chi-square statistic divided by cohort size	Summarizes association strength as an effect size independent of sample size

Preparation of a future validation dataset

A subset of reports was prepared for potential future validation of pipeline performance. This reference dataset was not used for evaluation in the current technical implementation report. A total of 400 reports were sampled to include all four possible combinations of the two binary classification labels. Two files were generated: (i) a review file containing only report text and report identifiers, with pipeline labels withheld, and (ii) a separate key file linking each identifier to the corresponding pipeline labels and reviewer annotations. This separation allows the review file to be redistributed or independently rescored without exposing the pipeline classifications, facilitating future validation studies.

Technical factors affecting output consistency

Several aspects of this deployment are worth noting explicitly as configuration-dependent factors, distinct from the pipeline's classification accuracy. The quantized weight format served under the Qwen2.5:7b-instruct tag is set by the Ollama runtime rather than pinned by this pipeline, and default quantizations distributed under a given tag can change across Ollama releases [11]; a party seeking to match this configuration exactly should record and pin both the Ollama version and the specific model digest, not only the tag name. Sampling parameters such as temperature were left at runtime defaults rather than fixed in the request payload, which leaves open the possibility of small run-to-run output variation even on identical input, though this was not formally quantified. Finally, because batches are dispatched concurrently and collected as they complete, the order in which reports are processed varies from run to run; this does not affect the final classification assigned to any individual report, since results are merged by report identifier rather than by completion order, but it does mean that log output and processing time are not themselves deterministic across runs.

## Discussion

The purpose of this report is to describe, at the implementation level, how a locally deployed Qwen2.5-7B-Instruct pipeline for structured extraction of musculoskeletal radiology report features was built and configured, including its prompt specification, parsing and retry behavior, and runtime environment. Several published evaluations of LLM-based radiology extraction report aggregate performance metrics while providing only general descriptions of prompting strategies, making it difficult to determine how much observed performance depends on model selection versus task-specific prompt engineering [3,12,13]. By documenting the complete prompt terminology, output-processing workflow, and deployment configuration, this report provides a transparent technical reference for readers seeking to evaluate, reproduce, or extend similar pipelines.

More generally, the choice of positive-evidence terminology described in the prompt specification directly shapes the pipeline's error behavior. The common extensor terminology given above includes several lower-specificity descriptors, such as general abnormality and irregularity, that may be satisfied by minor or incidental findings not intended to represent clinically meaningful pathology, and that a reader evaluating or adapting this pipeline should weigh accordingly. Because the exact terminology and negation rules are given in full above, a group extending or revising this prompt has a concrete starting point for testing narrower terminology or a tighter negation rule set for a given variable, and for assessing how such changes affect downstream labeling behavior and, in future validation studies, their impact on false-positive and false-negative classifications.

The configuration factors described above, comprising unpinned quantization, unfixed sampling parameters, and non-deterministic processing order, are not unique to this pipeline; they are generic properties of locally served, open-weight LLM deployments that are seldom discussed in application-focused papers. We describe them here deliberately, rather than treating the pipeline's output as if it were generated under fully controlled conditions, because a reader trying to understand this pipeline's behavior in detail should know which configuration values were fixed by design and which were left at framework defaults.

More broadly, this report reflects a growing recognition that published descriptions of LLM-based radiology tools should extend beyond aggregate performance metrics to include the operational specification of the pipeline itself. Reporting checklists proposed for artificial intelligence in medical imaging have historically emphasized dataset characteristics, model architecture, and validation design, but locally served, open-weight LLM pipelines introduce a further layer of configuration, spanning runtime version, quantization, sampling parameters, and prompt content, that existing checklists do not consistently capture [14]. As local deployment becomes more common for privacy-sensitive extraction tasks, a technical description of this kind may be useful not only as a record of a single pipeline but as an example of the level of operational detail such descriptions can include.

This report has limitations. It describes one specific pipeline configuration and does not include a systematic comparison against alternative prompt designs, alternative model sizes within the Qwen2.5 family, or a rule-based extraction baseline, so it should not be read as evidence that this particular configuration is optimal, only that it is fully specified. Extending this description to include a containerized environment definition with pinned dependency and runtime versions would further support exact replication of this configuration and is a natural next step.

## Conclusions

This report describes the implementation of a locally deployed Qwen2.5-7B-Instruct pipeline for structured classification of musculoskeletal radiology reports. It details the software environment, cohort-definition logic, prompt construction, model execution workflow, output parsing and failure-handling procedures, and downstream data-processing framework used to transform free-text radiology reports into structured variables. The goal of this technical report is to provide a reproducible description of the pipeline architecture and operational behavior, allowing readers to understand its design, evaluate its implementation choices, and adapt similar workflows for related clinical text-extraction applications without requiring access to the original codebase.

Because this work focuses on technical implementation, the generated classifications should be interpreted as automated pipeline outputs rather than validated clinical determinations. Formal assessment of classification performance, agreement with expert interpretation, and clinical utility requires independent validation against a reference standard and represents a separate future evaluation step. We additionally describe deployment-specific factors, including model serving configuration, quantization considerations, sampling parameters, and batch-processing behavior, as these details influence pipeline operation and are essential for accurately reproducing or extending locally deployed language model workflows.
